# American Medical Association

**Published:** 1851-10

**Authors:** 


*American Medical Association. Prize Essays.* — At a meeting of the American Medical Association, held in Charleston, S. C., in May last, the undersigned were appointed a committee to receive and examine such voluntary communications on subjects connected with medical science as individuals might see fit to make, and to award a prize to any number of them not exceeding five, if they should be regarded as entitled to such a distinction.

To carry into effect the intentions of the association, notice is hereby given that all such communication must be sent, post-paid, on or before the first day of April, 1852, to Geo. Hayward, M. D., Boston, Mass. Each communication must be accompanied by a sealed packet containing the name of the author, which will not be opened unless the accompanying communication be deemed worthy of a prize. The authors of the unsuccessful papers may receive them on application to the committee, at any time after the first of June, 1852; and the successful ones, it is understood, will be printed in the Transactions of the Association.

GEORGE HAYWARD, *Boston.*
J. B. S. JACKSON, "
D. H. STORER, "
JACOB BIGELOW, "
USHER PARSONS, *Providence, R. I.*

BOSTON, Aug. 20th, 1851.

